# Supplementary material for: Feature similarity gradients detect alterations in the neonatal cortex associated with preterm birth
Source: Hum Brain Mapp. 2024 Mar 15;45(4):e26660. doi: 10.1002/hbm.26660 (PMC10941526; doi:10.1002/hbm.26660)
Supplement: Supplementary file 1 — Data S1. Supporting Information. [file HBM-45-e26660-s001.pdf]

Appendix A. Statistical comparison of cortical metrics between datasets

We compared cortical thickness, curvature, surface areas and sulcal depth of the reconstructed cortical surfaces between the TEBC and the dHCP dataset using PALM. Before running the comparison, cortical maps were smoothed with a 4mm kernel. Prematurity, age at scan and sex were included as covariates of no interest. Permutation p-values were computed over 10000 random shuffles with threshold-free cluster enhancement and family-wise error rate corrections, and statistical significance was set at $p < 0.0253$ (equivalent to $\alpha = 0.05$, after Šidák correction over the two hemispheres). For cortical thickness (Fig. A.11), differences were located primarily in the medial part of the cortical surfaces, including medial portion of the frontal and posterior temporal cortices, and in the parietal cortices, where cortical thickness was higher in the TEBC data. In contrast, higher thickness values in the dHCP dataset were found in the superior frontal gyrus and orbito-frontal cortex, the temporal pole and the medial occipital cortex. Differences in curvature were less widespread, and located in correspondence of gyri (Fig. A.12). On the contrary, differences in surface area covered almost the entirety of the cortex, with values always higher in the dHCP dataset. Finally, higher sulcal depth values were observed in the TEBC dataset in the medial portions of the fronto-parietal cortices, the occipital lobe, the temporal lobe and in correspondence of the precentral and postcentral gyri, while the insular cortex, the medial orbitofrontal cortex and the superior temporal and the occipito-temporal gyri showed higher sulcal depth values in the dHCP dataset. In conclusion, there was no clear pattern across metrics that could suggest systematic differences attributable to varying quality of cortical reconstruction between the two datasets.

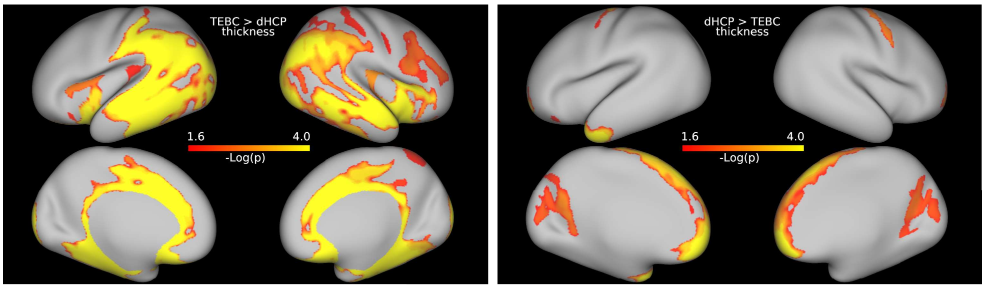

Figure A.11: P-value maps showing the regions where cortical thickness was higher in the TEBC sample (left) and in the dHCP sample (right), after controlling for prematurity, age at scan and sex.

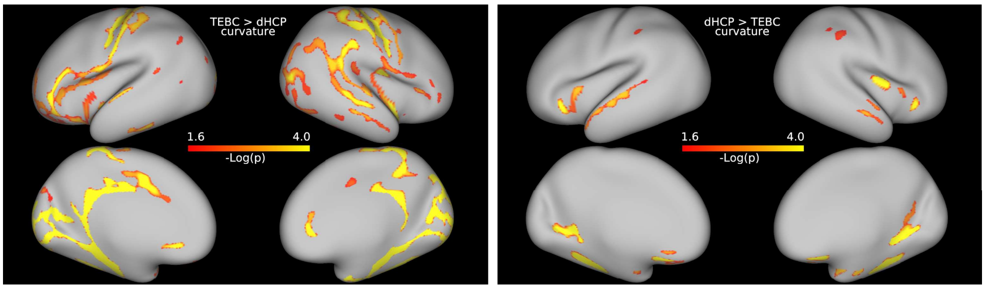

Figure A.12: P-value maps showing the regions where local curvature was higher in the TEBC sample (left) and in the dHCP sample (right), after controlling for prematurity, age at scan and sex.

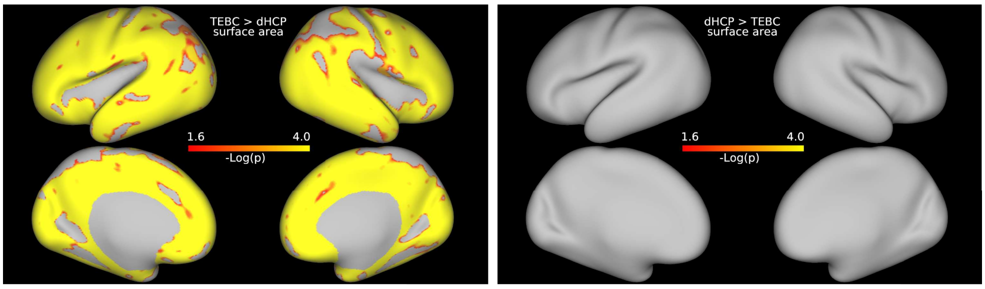

Figure A.13: P-value maps showing the regions where surface area was higher in the TEBC sample (left) and in the dHCP sample (right), after controlling for prematurity, age at scan and sex.

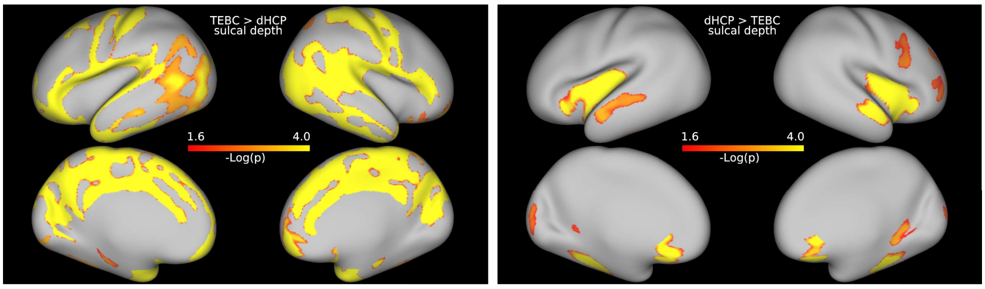

Figure A.14: P-value maps showing the regions where sulcal depth was higher in the TEBC sample (left) and in the dHCP sample (right), after controlling for prematurity, age at scan and sex.

Appendix B. Statistical comparison of microstructural maps between datasets

To further investigate the variability of results across datasets, we designed an additional experiment to compare microstructural maps between the TEBC and the dHCP dataset. To this end, subject metric maps in volumetric native space were projected onto individual cortical surfaces. The obtained surface maps were smoothed and registered to the 40-week dHCP cortical template before running a vertex-wise dataset comparison in PALM, controlling for prematurity, age at scan and sex. As for the other experiments, permutation p-values were computed over 10000 random shuffles with threshold-free cluster enhancement and family-wise error rate corrections, and statistical significance was set at $p < 0.0253$ (equivalent to $\alpha = 0.05$, after Šidák correction over the two hemispheres). Results are shown in figures B.15 to B.20. Differences were distributed throughout the cortex for all the metrics. MD, AD and RD showed similar patterns, with higher values for the TEBC dataset in the temporal and occipital lobes, and higher values for the dHCP dataset in the fronto-parietal and insular cortex. FA maps showed higher values in the TEBC dataset in the medial prefrontal, cingulate and anterior temporal cortices, while in the dHCP dataset FA values were higher in the occipital and parietal lobe and posterior temporal cortex. In the NDI and ODI maps, the TEBC dataset showed higher values in the cingulate and medial fronto-pariental cortices and the right lateral prefrontal cortex, and the dHCP dataset showed higher values in the occipital, parietal and temporal lobes and the left frontal cortex.

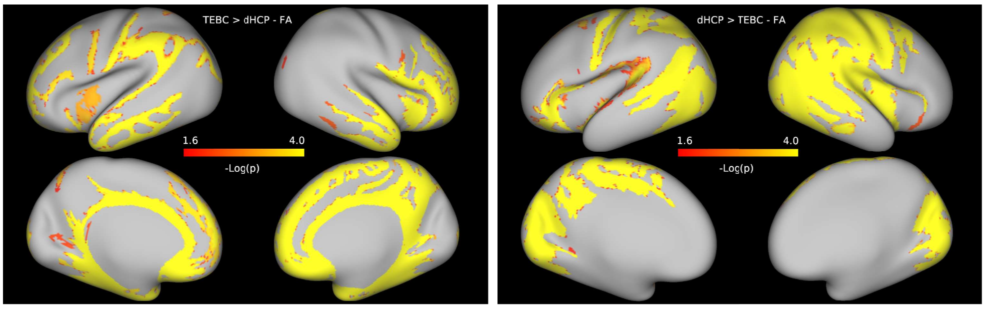

Figure B.15: P-value maps showing the regions where FA was higher in the TEBC sample (left) and in the dHCP sample (right), after controlling for prematurity, age at scan and sex.

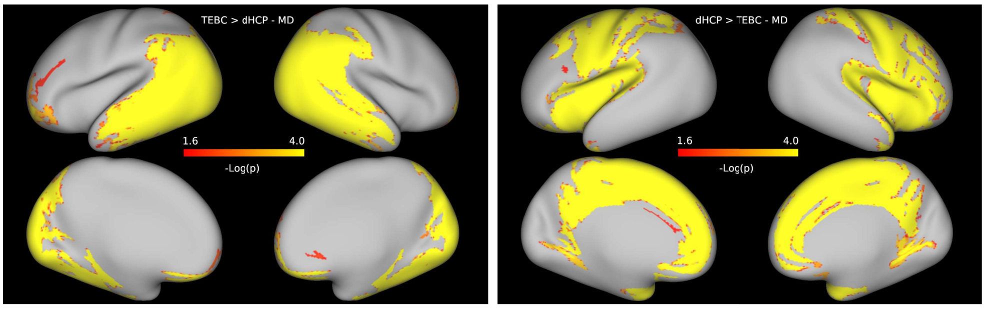

Figure B.16: P-value maps showing the regions where MD was higher in the TEBC sample (left) and in the dHCP sample (right), after controlling for prematurity, age at scan and sex.

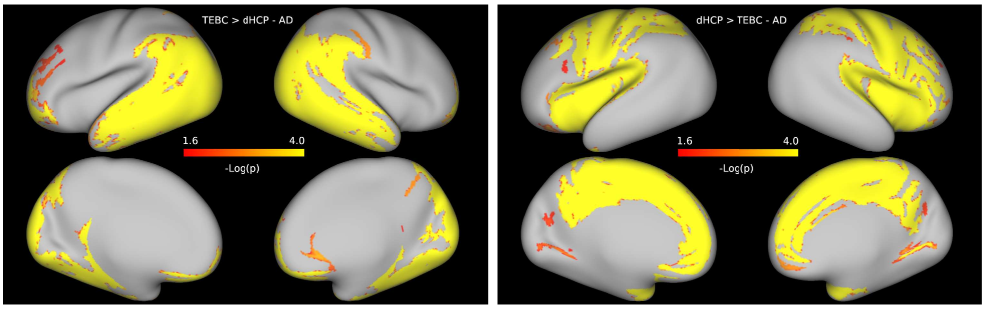

Figure B.17: P-value maps showing the regions where AD was higher in the TEBC sample (left) and in the dHCP sample (right), after controlling for prematurity, age at scan and sex.

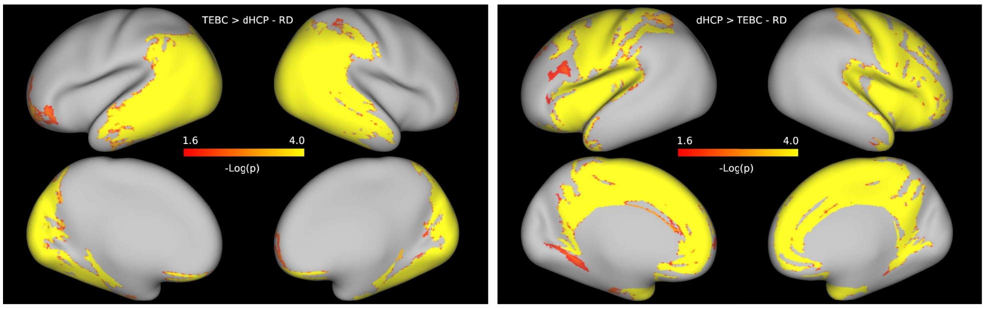

Figure B.18: P-value maps showing the regions where RD was higher in the TEBC sample (left) and in the dHCP sample (right), after controlling for prematurity, age at scan and sex.

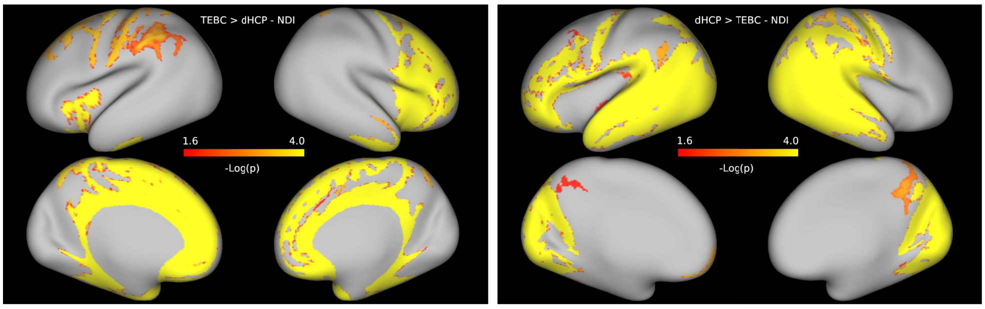

Figure B.19: P-value maps showing the regions where NDI was higher in the TEBC sample (left) and in the dHCP sample (right), after controlling for prematurity, age at scan and sex.

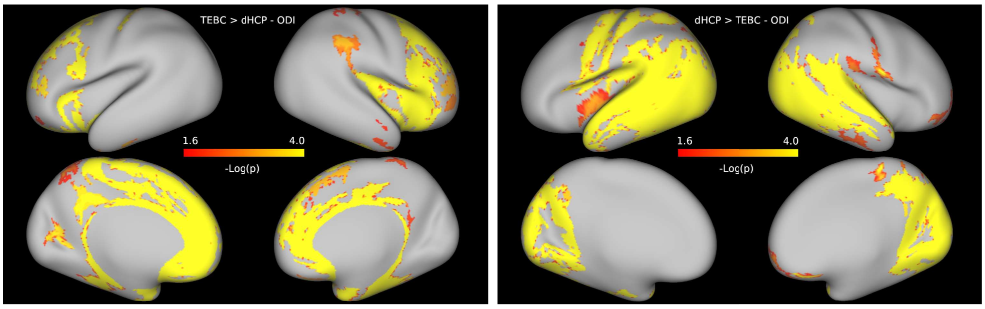

Figure B.20: P-value maps showing the regions where ODI was higher in the TEBC sample (left) and in the dHCP sample (right), after controlling for prematurity, age at scan and sex.
